# Association of Childhood Economic Hardship with Adult Height and Adult Adiposity among Hispanics/Latinos. The HCHS/SOL Socio-Cultural Ancillary Study

**DOI:** 10.1371/journal.pone.0149923

**Published:** 2016-02-26

**Authors:** Carmen R. Isasi, Molly Jung, Christina M. Parrinello, Robert C. Kaplan, Ryung Kim, Noe C. Crespo, Patricia Gonzalez, Natalia A. Gouskova, Frank J. Penedo, Krista M. Perreira, Tatiana Perrino, Daniela Sotres-Alvarez, Linda Van Horn, Linda C. Gallo

**Affiliations:** 1 Department of Epidemiology & Population Health, Albert Einstein College of Medicine, Bronx, NY, United States of America; 2 School of Nutrition and Health Promotion, Arizona State University, Phoenix, AZ, United States of America; 3 Graduate School of Public Health, San Diego State University, San Diego, CA, United States of America; 4 Collaborative Studies Coordinating Center, Department of Biostatistics, Gillings School of Global Public Health, University of North Carolina at Chapel Hill, Chapel Hill, NC, United States of America; 5 Department of Medical Social Sciences, Feinberg School of Medicine, Northwestern University, Chicago, IL, United States of America; 6 Department of Public Policy, University of North Carolina at Chapel Hill, Chapel Hill, NC, United States of America; 7 Department of Public Health Sciences, Miller School of Medicine, University of Miami, Miami, FL, United States of America; 8 Department of Preventive Medicine, Feinberg School of Medicine, Northwestern University, Chicago, IL, United States of America; 9 Department of Psychology, San Diego State University, San Diego, CA, United States of America; University of Oxford, UNITED KINGDOM

## Abstract

The study examined the association of childhood and current economic hardship with anthropometric indices in Hispanic/Latino adults, using data from the HCHS/SOL Socio-cultural ancillary study (N = 5,084), a community-based study of Hispanic/Latinos living in four urban areas (Bronx, NY, Chicago, IL, Miami, FL, and San Diego, CA). Childhood economic hardship was defined as having experienced a period of time when one’s family had trouble paying for basic needs (e.g., food, housing), and when this economic hardship occurred: between 0–12, 13–18 years old, or throughout both of those times. Current economic hardship was defined as experiencing trouble paying for basic needs during the past 12 months. Anthropometry included height, body mass index (BMI), waist circumference (WC), and percentage body fat (%BF). Complex survey linear regression models were used to test the associations of childhood economic hardship with adult anthropometric indices, adjusting for potential confounders (e.g., age, sex, Hispanic background). Childhood economic hardship varied by Hispanic background, place of birth, and adult socio-economic status. Childhood economic hardship during both periods, childhood and adolescence, was associated with shorter height. Childhood economic hardship was associated with greater adiposity among US born individuals only. Current economic hardship was significantly associated with all three measures of adiposity (BMI, WC, %BF). These findings suggest that previous periods of childhood economic hardship appear to influence adult height more than adiposity, whereas current economic hardship may be a better determinant of adult adiposity in Hispanics.

## Introduction

Studies examining social inequalities as determinants of health demonstrate that early socio-economic disadvantage contributes to the development of obesity and chronic diseases later in life. [1–7] Most of the studies reporting an inverse association of childhood socio-economic status (SES) and adult obesity have been conducted in developed societies.[8] In developing countries, this association appears to be reversed, with studies reporting greater obesity among individuals of higher SES.[1] A systematic review of childhood socio-economic position and waist circumference found that childhood poverty was associated with greater waist circumference, but adjustment for current SES weakened these associations.[9] In previous studies, childhood SES has been assessed in different ways (e.g. parental educational attainment) but few studies have used measures of economic hardship. Being raised in poverty may be a stronger predictor of adult adiposity since it is related to malnutrition during the intrauterine period and infancy, which can shape hormonal and physiological responses to richer diets later in life.[10,11] Furthermore, it has been reported that economic hardship during childhood may affect adult height due to malnutrition during early childhood.[10]

There is scarce information about relationships between childhood poverty and adult anthropometric traits in minority and immigrant populations living in developed societies.[8] Therefore, in this study we examined whether childhood and current adult economic hardship is associated with adult obesity in a sample of Latinos living in the US. We also examined adult height and other measures of adiposity (percentage body fat and waist circumference), as there is some evidence that the physiological effects of malnutrition early in life predisposes to short stature and the accumulation of abdominal fat.[10]

## Materials and Methods

HCHS/SOL is a population-based cohort study of 16,415 Hispanic/Latino adults (ages 18–74 years) who were selected using a two-stage probability sampling design from four US communities (Chicago, IL; Miami, FL; Bronx, NY; San Diego, CA). The HCHS/SOL Socio-Cultural Ancillary Study (SCAS) was conducted to examine the role of socio-economic and psychosocial factors on cardiovascular health. This study enrolled 5,313 participants from the HCHS/SOL between February 2010 and June 2011. Participants were asked to return to the HCHS/SOL clinic within 9 months of their baseline exam to complete a comprehensive set of psychosocial measures. Details about the aims and methodology of HCHS/SOL and HCHS/SOL SCAS are published elsewhere.[12–14] Of the 5,313 participants, 229 were excluded because they were missing body mass index (n = 11), childhood poverty (n = 67) or adult current poverty measures (n = 151), leaving a final analytic sample of N = 5,084. The study was conducted with the approval of the Institutional Review Boards (IRBs) of Albert Einstein College of Medicine, Feinberg School of Medicine at Northwestern University, Miller School of Medicine at the University of Miami, San Diego State University, and University of North Carolina at Chapel Hill. Written informed consent was obtained from all study participants.

### Measures

#### Anthropometric indices

Height (cm) was measured with a wall stadiometer (SECA 222, Germany) and weight (Kg) was obtained with a digital scale (Tanita Body Composition Analyzer, TBF 300, Japan). Body mass index (BMI) was calculated as weight in kilograms divided by height in meters squared. Waist circumference (cm) was obtained using the lateral border of the ilium as the anatomical reference, according to a standardized protocol. Percentage body fat was obtained by bioelectrical impedance analysis using the Tanita Body Composition Analyzer (TBF 300, Japan).

#### Childhood economic hardship

Participants were asked to report whether their families ever experience a period of time when they had trouble paying for their basic needs, such as food, housing, medical care, or utilities, when they were a child. Those participants who answered “Yes” to the previous question were asked to report whether the hardship occurred between the ages of 0–12 years old or 13–18 years old. Using this information, the following categories were created:

1. Did not experience childhood economic hardship
2. Economic hardship during childhood (0–12 years old) only
3. Economic hardship during adolescence (13–18 years old) only
4. Economic hardship during both periods, childhood and adolescence.

#### Current economic hardship

Participants were asked to report whether they experienced trouble paying for basic needs such as food, housing, medical care, or utilities in the last 12 months.

#### Socio-demographic variables

Participants also reported their Hispanic/Latino background (Cuban, Dominican, Mexican, Puerto Rican, Central or South American, and other/mixed), date of birth, sex, place of birth (born within the 50 US states or not), years living in the US, annual household income and educational attainment.

### Statistical analysis

Differences in childhood economic hardship by adult socio-demographic (e.g. sex, age, income) characteristics were evaluated using the Rao-Scott chi-square test. To assess the association of childhood economic hardship with adult anthropometric indices (height, BMI, waist circumference, and body fat), separate survey linear regression models were fit, adjusting for age, sex, educational achievement, household income, Hispanic/Latino background, field center, and years of living in the US. We tested for interactions by sex, age, place of birth, and age of immigration in the association of childhood economic hardship and anthropometric indices by adding a product term to the models (e.g. childhood economic hardship*sex). When the interactions were significant, results were presented stratified. In addition, the association of current (adult) economic hardship with adiposity measures was also examined using survey linear regression models adjusted for the aforementioned variables. Analyses were weighted to account for the complex sampling design and nonresponse using SAS-callable SUDAAN version 11.0 (SAS Institute, Cary, NC).

## Results

The study sample included 3,163 women and 1,921 men. The mean (SD) age was 46.4 (13.7) years, with 3,117 (61.3%) ≥45 years old. Participants were predominantly born outside of the 50 US mainland states (82.7%) and were of low SES: 36.3% did not graduate from high school; 48.4% had annual household income ≤ $20,000. About half of participants (53.8%) reported currently experiencing economic hardship). Socio-demographic characteristics (sex, place of birth, educational achievement, and annual household income were similar between the included and excluded groups. However, the percentage of older adults (>45 years old) was higher in participants excluded from the analyses (70% vs. 61%, p value = 0.006). Fifty three percent of participants reported that their families had difficulties in paying for basic needs when they were children. Of those reporting childhood economic hardship, 25% reported that this economic hardship occurred during childhood only, 7% during adolescence only and 68% reported that this economic hardship occurred during childhood and adolescence.

### Height

A multivariate model, adjusted for age, sex, Hispanic background, household income, educational attainment, field center and study design, showed that men were taller than women (β = 13.22, 95% confidence interval: 12.69, 13.74), and that there was an inverse association of age with height (β = -0.10 95% confidence interval: -0.12, -0.08). Individuals of Dominican (β = 4.08, 95% confidence interval: 2.75, 5.42), Puerto Rican (β = 2.98, 95% confidence interval: 1.82, 4.15) and Cuban background (β = 2.56, 95% confidence interval: 1.14, 3.99) were taller than individuals of Mexican heritage. The height of individuals of Central (β = -0.15, 95% confidence interval: 1.54, 1.23) and South American background (β = 0.77, 95% confidence interval: -0.64, 2.18) was not statistically significant different from individuals of Mexican background. Individuals born outside the US 50 states were significantly shorter than those born within the 50 states (β = -1.43, 95% confidence interval: -2.18, -0.68). This model also showed that participants who reported economic hardship during both childhood and adolescence were significantly shorter than those reporting not experiencing childhood poverty (β = -0.60, 95% confidence interval: -0.17, -0.04). Stratified analysis by sex (Table 1) indicated that the association between childhood economic hard ship and shorter stature was observed only in men (p for interaction = 0.0065). There was no interaction with place of birth or age at immigration.

**Table 1:** Multivariate survey linear regression models for the association of childhood economic hardship with adult anthropometric indices stratified by sex^†^.

	Height (cm)		BMI (kg/m2)		WC (cm)		Body fat (%)	
	β	95% CI	β	95% CI	β	95% CI	β	95% CI
**Women (n = 3,091)**								
No economic hardship	Ref		Ref		Ref		Ref	
During childhood only	-0.11	-1.00, 0.77	-0.67	-1.69, 0.36	-2.2	-4.37, -0.03	-1.26	-2.59, 0.07
During adolescence	0.66	-0.68, 2.00	-1.1	-2.68, 0.47	-1.98	-5.83, 1.87	-1.33	-3.45, 0.80
During childhood and adolescence	0.18	-0.47, 0.83	0.03	-0.83, 0.90	0.43	-1.10, 1.96	0.17	-0.66, 0.99
**Men (n = 1,890)**								
No economic hardship	Ref		Ref		Ref		Ref	
During childhood only	0.3	-0.90, 1.49	0.94	-0.13, 2.02	2.35	-0.38, 5.08	1.75	0.15, 3.35
During adolescence	-0.08	-1.78, 1.63	0.5	-1.48, 2.47	2.23	-3.00, 7.45	-0.52	-3.20, 2.15
During childhood and adolescence	-1.51	-2.41, -0.61	0.41	-0.40, 1.22	0.55	-1.50, 2.60	0.88	-0.37, 2.13

^†^Adjusted for age, background, field center, education, household income, and years in US

### Adiposity Measures

Multiple linear regression models adjusting for potential confounders showed that men had a lower BMI (β = -1.20; 95% confidence interval: -1.67, -0.73) and percentage body fat (β = -10.92; 95% confidence interval: -11.57, -10.26) but higher waist circumference (β = 2.15; 95% confidence interval: 0.92, 3.37) than women. Compared to individuals of Mexican background, participants of Puerto Rican origins had a higher BMI (β = 1.59; 95% confidence interval: 0.49, 2.69), but percentage body fat and waist circumference did not vary by Hispanic/Latino background. Overall, childhood economic hardship was not associated with adiposity measures. However, in stratified analysis we found that among individuals US born individuals childhood economic hardship during childhood and adolescence was associated with higher BMI, waist circumference and percentage body fat (Table 2), associations that were not observed in foreign-born individuals (p for interaction = 0.0378 for BMI, 0.0672 for waist circumference and 0.22 for percentage body fat). No interaction was observed with age, sex or age at immigration.

**Table 2:** Multivariate survey linear regression models for the association of childhood economic hardship with adult anthropometric indices stratified by place of birth^†^.

	Height (cm)		BMI (kg/m2)		WC (cm)		Body fat (%)	
	β	95% CI	β	95% CI	β	95% CI	β	95% CI
**Born within US 50 states (n = 866)**								
No economic hardship	Ref		Ref		Ref		Ref	
During childhood only	0.35	-1.05, 1.74	0.97	-1.01, 2.96	1.75	-2.97, 6.47	0.99	-1.98, 3.95
During adolescence only	0.63	-1.57, 2.82	1.33	-0.99, 3.65	4.18	-1.88, 10.24	1.38	-2.35, 5.10
During childhood and adolescence	-0.31	-1.66, 1.05	1.68	0.06, 3.31	3.73	0.08, 7.38	2.21	0.14, 4.29
**Born outside US 50 states**†† **(n = 4,115)**								
No economic hardship	Ref		Ref		Ref		Ref	
During childhood only	0.09	-0.78, 0.97	-0.31	-1.12, 0.49	-0.79	-2.64, 1.06	-0.16	-1.22, 0.90
During adolescence only	0.12	-1.07, 1.31	-1.11	-2.37, 0.16	-2.06	-5.20, 1.07	-1.67	-3.28, -0.06
During childhood and adolescence	-0.66	-1.23, -0.09	-0.3	-0.90, 0.29	-0.65	-2.00, 0.70	-0.06	-0.87, 0.74

^†^ Adjusted for age, sex, background, field center, education, household income, and years in US (for FB only).

^††^Includes those born in US territories, such as Puerto Rico

In addition, individuals reporting current economic hardship had higher BMI (β = 1.17; 95% confidence interval: 0.69, 1.66), larger waist circumference (β = 2.52; 95% confidence interval: 1.41, 3.64) and higher percentage body fat (β = 1.41; 95% confidence interval: 0.72, 2.10), compared to those without these difficulties, independently of age, sex, educational achievement, household income, Hispanic background, years living in the US and study design. Further adjustment for childhood economic hardship slightly attenuated the magnitude of effects for BMI (β = 1.12; 95% confidence interval: 0.63, 1.60), waist circumference (β = 2.24; 95% confidence interval: 1.10, 3.38) and percentage body fat (β = 1.32; 95% confidence interval: 0.61, 2.04). The association between current economic hardship and adiposity measures were stronger among US-born group, compared to the foreign-born (p for interaction = 0.005 for BMI, 0.013 for waist circumference and 0.009 for percentage body fat) (Table 3). No interaction was observed with age, sex or age at immigration.

**Table 3:** Multivariate survey linear regression models for the association of current economic hardship with adult adiposity indices stratified by place of birth.

	BMI (kg/m2)		WC (cm)		Body fat (%)	
	β	95% CI	β	95% CI	β	95% CI
**Model 1**						
**Born outside US 50 states**						
No economic hardship in past 12 months	Ref		Ref		Ref	
Economic hardship in past 12 months	0.73	0.25, 1.21	1.61	0.49, 2.74	0.93	0.20, 1.66
**Born within US 50 states**						
No economic hardship in past 12 months	Ref		Ref		Ref	
Economic hardship in past 12 months	2.92	1.63, 4.21	6.2	3.26, 9.15	3.32	1.63, 5.01
**Model 2**						
**Born outside US 50 states**						
No economic hardship in past 12 months	Ref		Ref		Ref	
Economic hardship in past 12 months	0.78	0.27, 1.29	1.58	0.38, 2.78	0.95	0.17, 1.73
**Born within US 50 states**						
No economic hardship in past 12 months	Ref		Ref		Ref	
Economic hardship in past 12 months	2.32	0.98, 3.65	4.66	1.50, 7.82	2.51	0.72, 4.29

Model 1: adjusted for age, sex, background, education, income, and years in US (for FB only). Model 2: Model 2 + childhood deprivation

## Discussion

In this study childhood economic hardship was associated with adult shorter stature, and was more pronounced in men than in women. Prior studies examining the association of childhood socio-economic conditions and adult obesity have shown inconsistent results, with a few studies reporting that childhood poverty predicted obesity among women, but not in men.[15–17] In this study, we did not observe an interaction with sex, but there was a significant interaction with place of birth. There was an association between childhood economic hardship during childhood and adolescence with greater adiposity that was observed among US-born individuals only. Consistent with existing literature,[1,18] current socio-economic disadvantage was associated with significantly higher adiposity and this association was stronger among US-born participants. Economic hardship in adults may be associated with lower dietary quality and lack of opportunities for physical activity, which in turn could result in excess weight.[19,20] In addition, economic hardship could be associated with higher psychosocial stress, which has been shown to be associated with obesity.[21,22] Our findings indicate that childhood hardship attenuated the effects of current socio-economic conditions, which is consistent with the notion that childhood adversity could have long lasting implications, in particular if low socio-economic conditions persist.[23]

One important limitation of the study is the use of retrospective reports of childhood socio-economic conditions. About 50% of the sample reported having experienced economic hardship during childhood, and the majority reported hardship in both childhood and adolescence, suggesting persistent exposure to deprivation throughout the developing years. It is possible that individuals had limited ability to recall with accuracy the socio-economic conditions they grew up with at specific periods of their childhood; the fact that the majority of participants reported economic hardships during childhood and adolescents, and fewer reported hardship during one period alone. Furthermore, the study used a single question about family difficulties in paying for basic needs as a proxy for childhood economic hardship, and other relevant measures such as parental education was not assessed. Prospective studies that assessed childhood socioeconomic status at the moment of assembling the cohort, found that childhood socio-economic conditions predicted young adults’ weight status.[17] The positive association between childhood economic hardship and adiposity among US-born individuals could be explained by an environment characterized by limited access to healthy food and places to exercise, which is more predominant in low-income areas. An alternative explanation is the selective forces operating in the migration to the US, those who immigrated are postulated to be healthier and with economic resources, and possible other resilient factors, that made the migration possible.[24]

Short stature has been reported as a risk factor for chronic diseases;[25,26] but information about the correlates of adult height is scarce. In this sample of Hispanic/Latino adults, persistent childhood economic hardship was significantly associated with shorter stature, which could be related to poor nutrition during infancy and childhood. Data from middle and low income countries indicate that infant growth restriction is an important predictor of adult height.[27] Similarly, in countries where malnutrition is common, stunting predicted adult short stature.[28] [29] Other studies have also shown that height during infancy and childhood has a positive association with adult height.[30,31] It is interesting to note that in a Guatemalan cohort, stunting was not associated with adult adiposity.[28] The present study also found that being foreign born was associated with shorter stature, whereas individuals of Caribbean heritage were taller, compared to individuals of Mexican background. Consistent with our findings, an analysis of Mexican immigrants and Mexican American, born in the US, individual also found shorter stature among foreign-born.[24] As we did not collect information about early life characteristics such as nutritional status, we cannot address the specific factors that explain the observed associations with adult height. Nevertheless, these findings are intriguing and suggest that in addition to socio-economic conditions, genetic factors could be at play.[32]

Taken together, the study findings provide further evidence of the role of economic hardship on adult anthropometric indices. Whereas the study suggests that current socio-economic conditions are more relevant for adult adiposity, childhood economic hardship was associated with shorter stature. Shorter individuals have been found to be at increased risk of cardiovascular disease and other chronic conditions,[25,26] and recent evidence suggests that the genes that regulate height increase the risk of coronary disease.[33] Further studies are needed to help disentangle the interplay between genetic and socio-economic determinants of anthropometric traits, and the subsequent implications regarding the development of chronic diseases.
